# Removal of pectoral muscle based on topographic map and shape-shifting silhouette

**DOI:** 10.1186/s12885-018-4638-5

**Published:** 2018-08-01

**Authors:** Bushra Mughal, Nazeer Muhammad, Muhammad Sharif, Amjad Rehman, Tanzila Saba

**Affiliations:** 1 Department of Computer Science, COMSATS University Islamabad, Wah Campus, Wah Cantt, Pakistan; 2 Department of Mathematics, COMSATS University Islamabad, Wah Campus, Wah Cantt, Pakistan; 3 College of Computer and Information Systems, Al-Yamamah University, Riyadh, Saudi Arabia; 4 Department of Information Systems, Prince Sultan University, Riyadh, Saudi Arabia

**Keywords:** Mediolateral-oblique (MLO), Pectoral muscle, Breast profile, Cranial-caudal (cc), Label and artifacts

## Abstract

**Background:**

In digital mammography, finding accurate breast profile segmentation of women’s mammogram is considered a challenging task. The existence of the pectoral muscle may mislead the diagnosis of cancer due to its high-level similarity to breast body. In addition, some other challenges due to manifestation of the breast body pectoral muscle in the mammogram data include inaccurate estimation of the density level and assessment of the cancer cell. The discrete differentiation operator has been proven to eliminate the pectoral muscle before the analysis processing.

**Methods:**

We propose a novel approach to remove the pectoral muscle in terms of the mediolateral-oblique observation of a mammogram using a discrete differentiation operator. This is used to detect the edges boundaries and to approximate the gradient value of the intensity function. Further refinement is achieved using a convex hull technique. This method is implemented on dataset provided by MIAS and 20 contrast enhanced digital mammographic images.

**Results:**

To assess the performance of the proposed method, visual inspections by radiologist as well as calculation based on well-known metrics are observed. For calculation of performance metrics, the given pixels in pectoral muscle region of the input scans are calculated as ground truth.

**Conclusions:**

Our approach tolerates an extensive variety of the pectoral muscle geometries with minimum risk of bias in breast profile than existing techniques.

## Background

**Fig. 1:**
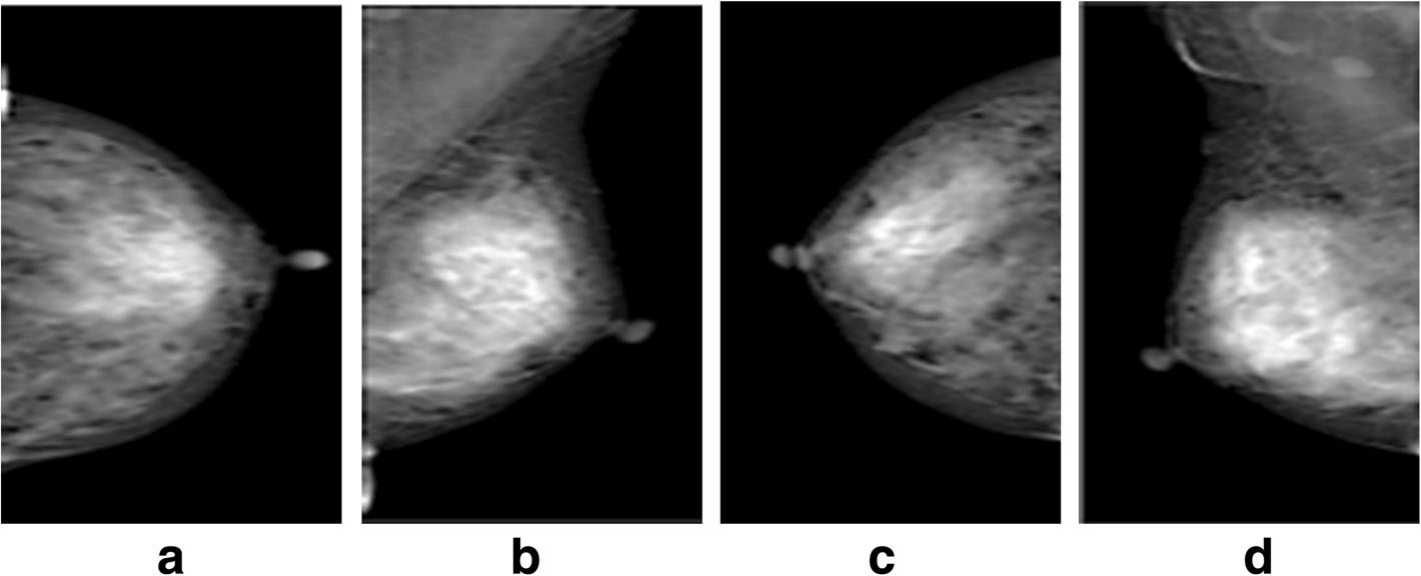
Two sided views of left and right mammograms: **a** left CC, **b** left MLO, **c** right CC and **d** right MLO

Breast cancer among women is a well-known disease throughout the world. About 1.68 million cases and the 522,000 deaths causes of the breast cancer were registered in 2012 [1]. Computer aided diagnosis (CAD) was designed to locate the premature level of the breast cancer [2]. A number of imaging techniques have also been presented to manage this issue, such as mammography [3], ultrasound [4], magnetic resonance imaging (MRI) [5], PET/CT scan [6], SPECT, thermogram [7], and tomography [8]. Mammography is one of the most suggested imaging modality to detect the breast tumor at early stage. In screening mammography [9], two different angels of breast body are stored in mammogram which are cranial-caudal (CC) and mediolateral-oblique (MLO) assessment as shown in Fig. 1.

CC is used to observe “top to bottom” information and MLO is used to observe the “side view”. The difficulty with the MLO view of mammogram is the larger area of the pectoral muscle mass tissue, complex contour, and structural volume. However, pectoral muscle is a dense region and prominent in mammogram. It does not provide any valuable information. Moreover, it also affects the segmentation, feature extraction, and classification process, which leads to the high rate of false positive.

In recent years, a lot of automatic pectoral muscle removal methods have been developed [10]. However, due to the variations in size, shapes, intensity, and contrasts of the pectoral muscles, most of the existing techniques [8–11] fail to remove accurate muscle regions from the entire mammograms. The advantages of our proposed method are: 1) muscle detection possibility is improved, even in low contrast problems, 2) pectoral muscle shape tracking is attained without using of the heuristic thresholding, and 3) to identify the boundary of a breast. The existence of these problems may lead to wrong assumptions of a false-(negative and positive) rates with un-desired biopsies [11].

The proposed work is arranged in following Sections. “Related work” shows a literature analysis of the existing approaches regarding pectoral muscle extraction process. “Proposed methodology” demonstrates the proposed method for approximating the skin line boundary for given breast body. “Experiments and results” provides the simulation results and discussion, whereas, conclusions are presented in “Conclusion”.

## Related work

Mammograms is known as a most recommended imaging modality to observe the breast cancer at initial stage [12]. The pectoral muscle in terms of mass tissue is used to support the breast body. Mostly pectoral muscle appears along with the breast tissues in Medio-Lateral Oblique (MLO) for observing the given mammograms. As a result segmentation data of the pectoral muscles with accurate contour by following the breast tissues has become challenging task in computer aided diagnosis (CAD) systems [13]. With existence of similarities in texture and pixel intensities of the pectoral muscles and breast tissues, it becomes very difficult to find out accurate region of interest or breast body which may lead towards awry CAD results. Usually, pectoral muscle is estimated in terms of a boundary measurement in form of straight line with range of an angle from 45° to 90°. Moreover, Hough transform (HM) was experienced to the accumulator cells for estimating a straight-line with the pectoral muscle of the given edges [14]. Another approach was used in order to find the pectoral muscle with the combination of the cliff detection technique and straight line estimation method [15]. An automatic procedure based on morphological operators and polynomial function is offered for finding pectoral muscles [16]. Various multi resolution techniques have been presented for extraction of the pectoral muscles [17]. A multi resolution approach is presented to classify the pectoral muscle of the MLO mammograms in wavelet domain [18]. A hybrid approach was presented to highlight the pectoral muscle and breast border using wavelet transform and bit depth reduction [19]. Pixel constancy constraint method is introduced at multi-resolution level for removing of the pectoral muscle [20]. Different techniques for locating the pectoral muscle edges based on contour detection and graphs have been discussed here. Combination of the minimum spanning trees and an active contour approach was presented for identifying the precise calculation of the pectoral muscle [21]. A method of the pectoral muscle identification at the rate of a 92% (DDSM database) and 90.06% (MIAS database) is presented in [22]. The method based on regression via RANSAC with edge detection have been proposed for contouring the muscle area [23]. Bezier curve method was established for leveling the region of the pectoral muscle using their control points [24]. An automated method based on normalized graph cuts segmentation technique is presented in [25]. Muscle contour detection method is adopted the shortest path with contour end point trained by support vector models [26].

A combination of an active contour technique is used with discrete time Markov chain (DTMC) for boundary detection of the pectoral muscle region. DTMC is determined to deal with two important properties of the pectoral muscle edges which are continuity and uncertainty. An active contour model is implemented on rough boundary to increase the detection rate of an affective part of the mammogram [27]. An intensity based approach with newly designed enhancement filter, and threshold method is presented to locate the contour of the pectoral muscle [28].

Various existing methods were demonstrated to extract the information of the pectoral muscle boundary [29–35]. Most of the techniques are constructed on the pixel divergences of the breast body and the tissues of the pectoral muscle. Intensity based segmentation methods may be noted using the intensity variations of a mass body tissues. However, it may cause an inconsistent segmentation outcome [29]. Recently, a number of the researchers tried to apply copious methods to achieve a sufficient segmentation rate using suitable intensity features [29–34]. With an exception of strong intensity based segmentation methods, histogram based founded techniques are conversed [14–16]. Furthermore, intensity based method designs by the hypothesis following the gray scale values with various structure of the known pectoral muscle could be achieved in higher order than its neighboring tissues [35–46].

## Methods

**Fig. 2:**
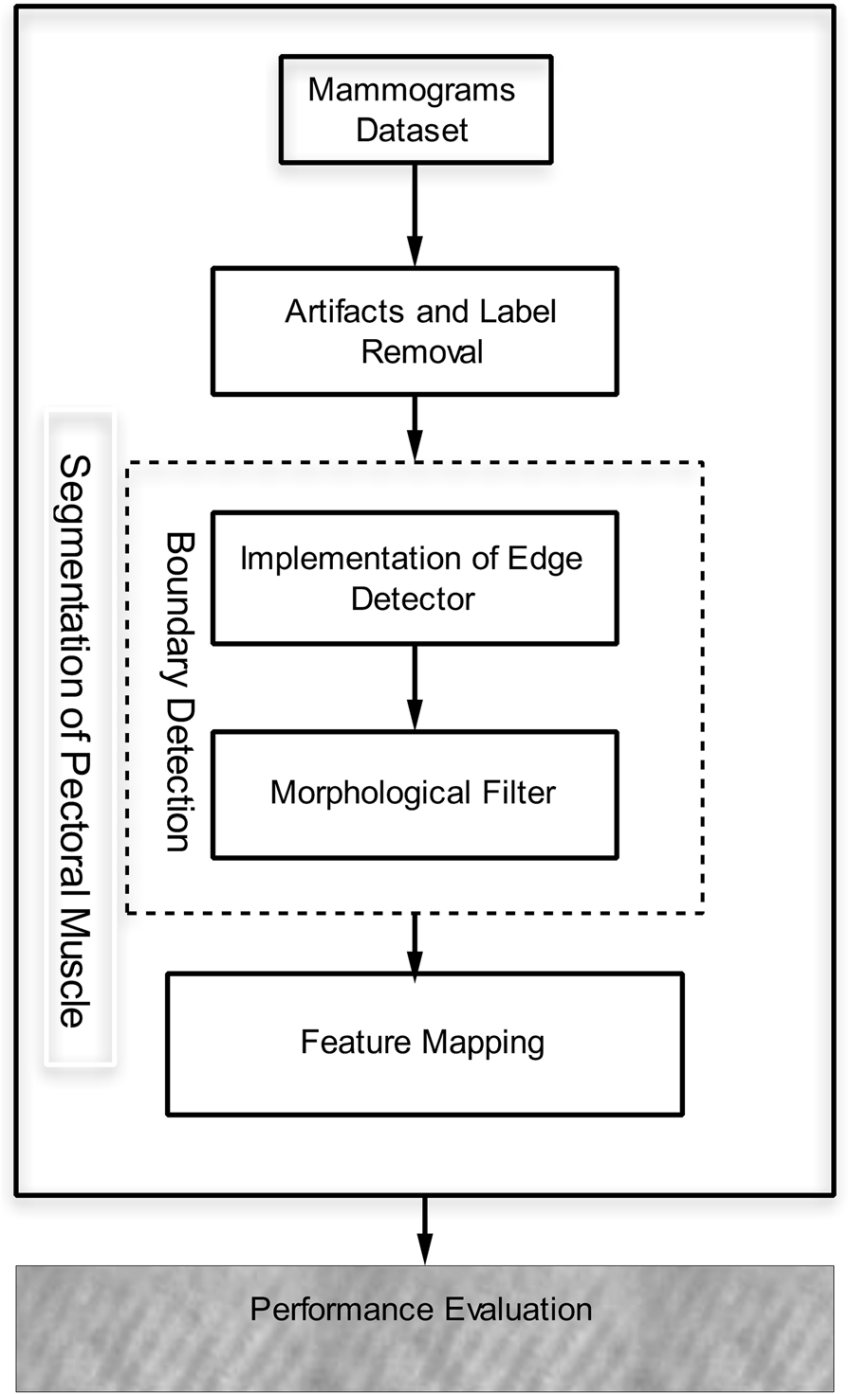
Proposed methodology

The input data taken in the proposed method is used from the benchmark dataset of the MIAS. These images may contain label and machine artifacts with high intensity value at the top. Let *P*_*ϱ*_ be the original mammogram on which segmentation is performed. In this regard, a flow chart is presented in Fig. 2.

### Segmentation of the pectoral muscle

**Fig. 3:**
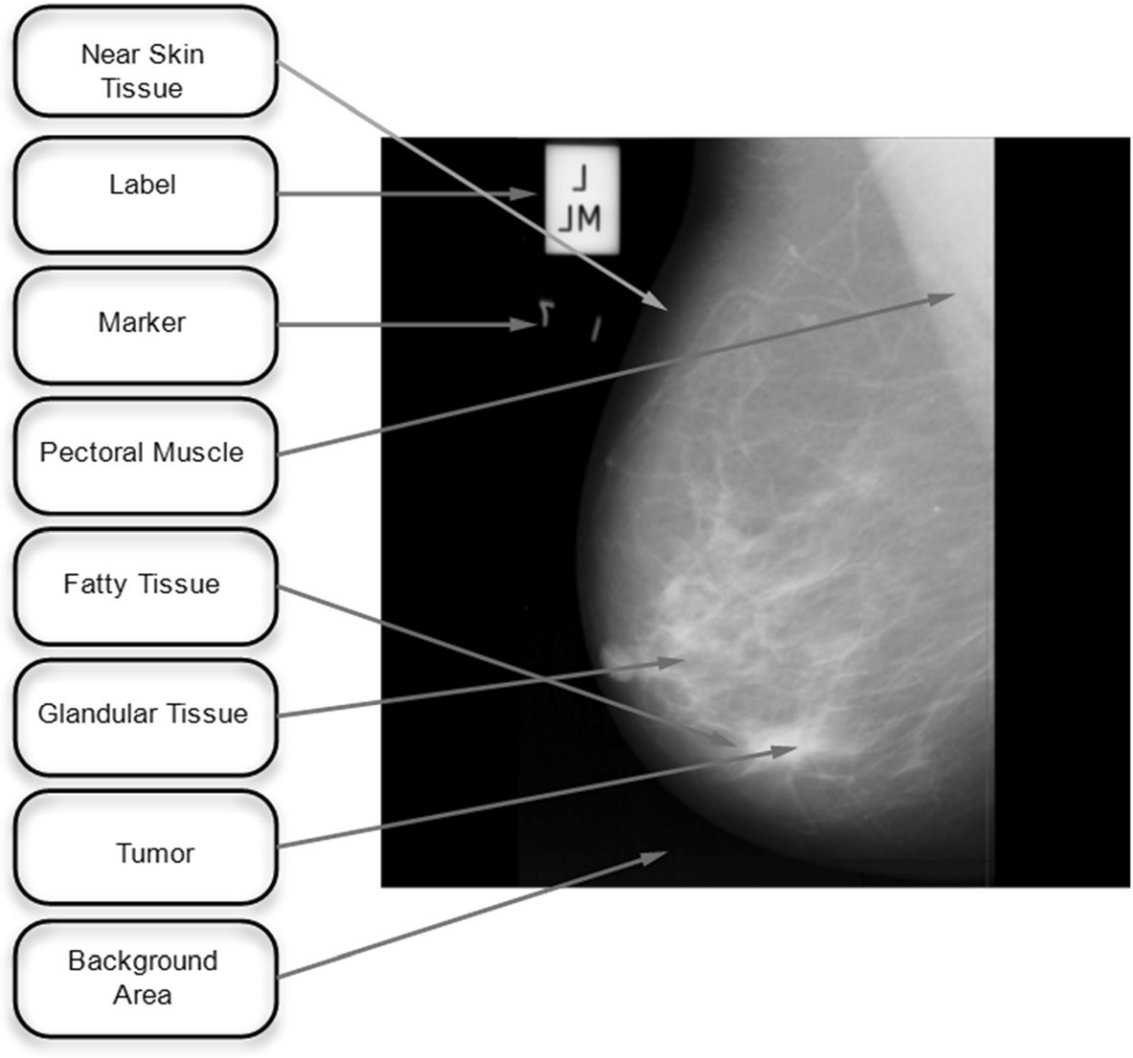
Labeled mammogram from mini MIAS

Our key drive of this research work was to elude the unnecessary areas from the breast region like pectoral muscle in a cost effective manner. Brightest pixels of the mammograms are present in the pectoral muscles regions. To avoid the false assumptions of positive results (mammogram shows cancer, but in fact there is no cancer), pectoral muscles regions should be removed, efficiently. Left or right pectoral muscles tissues are based on the front side view of the given mammogram. A labeled mammogram from the mini MIAS data is displayed in Fig. 3.

#### Label and artifacts removal

Usually background area in mammographic images may contain radiopaque artifacts, markers, and chocks. Let *f*(*∇*) be a label removal function applied on image *P*_*ϱ*_ which provides the binary image *I*_*κ*_ as shown in Eq. 1. *f*(*∇*) is used to remove the undesired labels by amplifying the areas of the high intensity and segment them using a seed. The seed point is initialized on the convex hull and erodes the map until it has converged on the edge of the areas to preserve the edge geometry as a result we get a binary image *I*_*κ*_as described in [46–51].

1 $$ {I}_{\kappa }={P}_{\varrho}\leftarrow f\left(\nabla \right). $$

2 $$ {I}_{\psi}\leftarrow {I}_{\kappa }. P\varrho $$

**Fig. 4:**
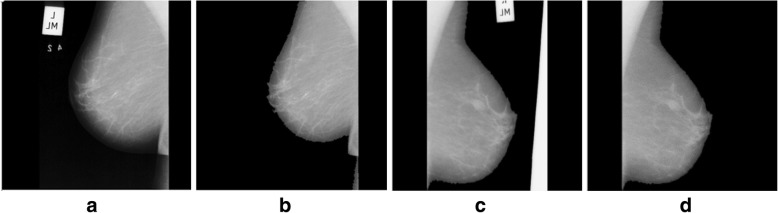
Label along with artifacts removal: **a** and **c** given mammogram (original) and (**b** and **d**) after label and artifact removal

Where *I*_*k*_ is used for preserving the original intensities to restore it back into gray scale (*I*_*ψ*_) image. The X-ray machine labels and certain other artifacts may be removed from the image and the object of interest is extracted as shown in Fig. 4.

#### Boundary detection

Boundary detection to suppress the pectoral muscle from a breast parenchyma is an important step of the proposed method. It is possible to recognize pectoral muscle within an image using mammography features in terms of the edge detection. To detect contours, the differential operator is often used in practice which includes isotropic, Sobel, and Prewitt operators. These operators compute the horizontal and vertical differences of the local sums with reduced noise effects. The pixel location (*α*, *β*) is declared an edge location if *φ*(*α*, *β*) exceeds some threshold 0 > *τ* < 1. A threshold value *τ* with range between 0 and 1 is used as a power feature. This is used to manage the scrambled edges.

The locations of the edge points constitute an edge map Ρ(*η*, *θ*) which is defined as

3 $$ \mathrm{P}\left(\eta, \theta \right)=\left\{\ \begin{array}{c}1,\kern1em \left(\alpha, \beta \right)\in {I}_{\varphi}\\ {}0,\kern4em else\end{array}\right.,\kern1.5em where\kern0.5em {I}_{\varphi }=\kern0.5em \left\{\left(\alpha, \beta \right);\kern0.5em \varphi \left(\alpha, \beta \right)>\tau \right\},\kern0.5em $$

**Fig. 5:**
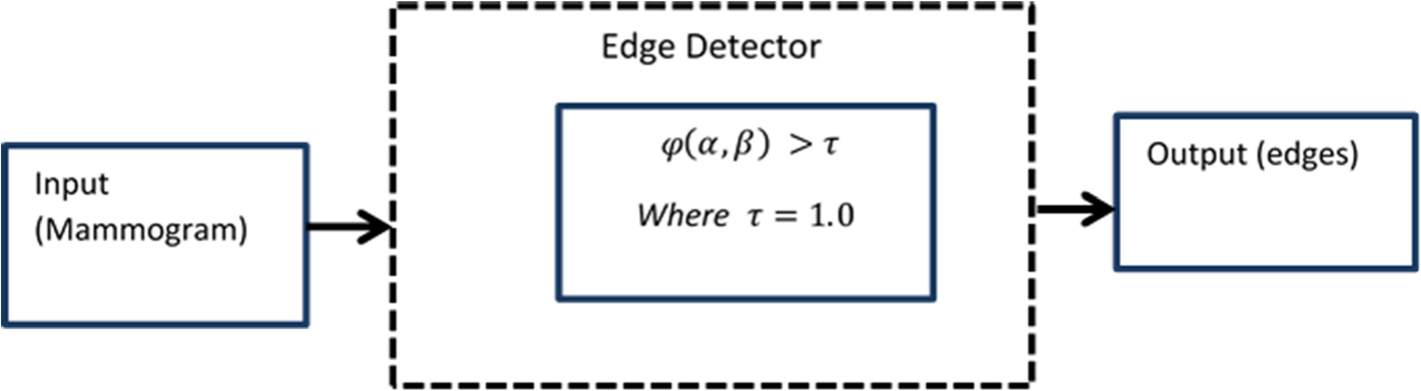
Edge detection map

**Fig. 6:**
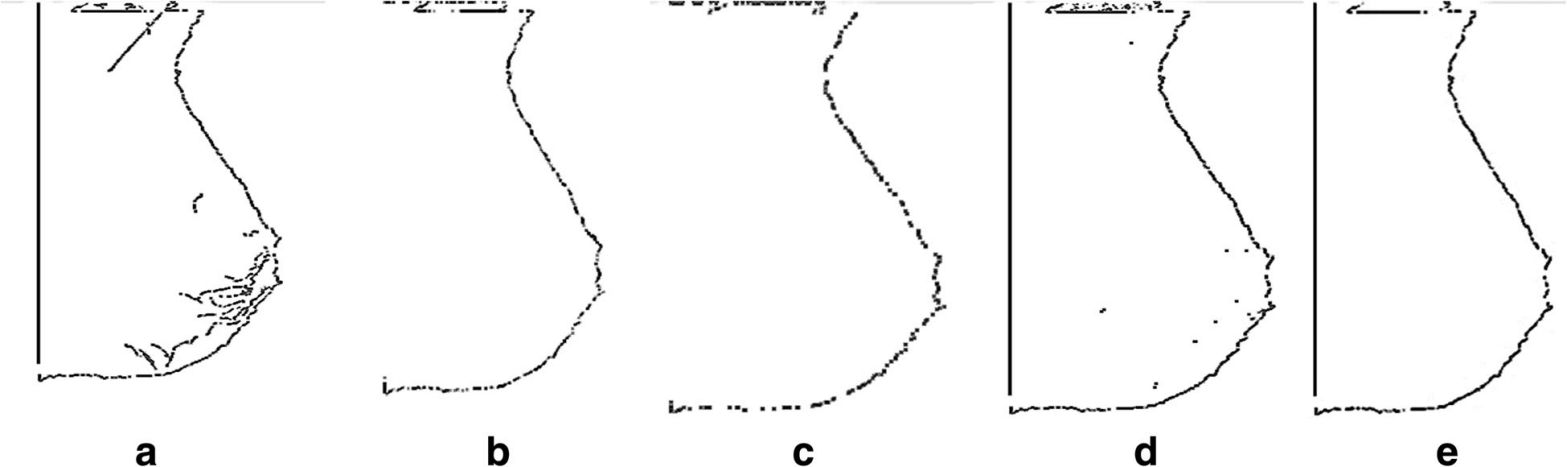
Detection of edges: **a** Canny, **b** Prewitt, **c** Sobel, **d** Robert and **e** Laplacian

The edge map provides the significant information regarding the boundaries in an image. Usually, threshold value *τ* may be selected using the accumulative histogram of *φ*(*α*, *β*) so that the pixels with largest gradients are represented as sharp edges. A general edge detector is presented in Fig. 5. Results of the various edge detectors are given below in Fig. 6.

The performance is observed in various edge detectors for analyzing the peak signal to noise ratio metric (PSNR), mean square error metric (MSE), and structural similarity index measurement metric (SSIM). All these measures are determined for quality assessment of mammographic image. Highest value of the PSNR and the SSIM with lower mean square error gives the best choice of the edge detector [52–58]. Performance measures of the various edge detectors on mammograms taken from the mini MIAS are given below in Table 1.

**Table 1:** Performance measures of the various edge detectors on mammograms

Algorithm	PSNR	SSIM	MSE
Prewitt	80.9174	0.718257820295515	6594.5601
Sobel	80.8861	0.718258704733238	6594.5573
Roberts	76.7231	0.718261446876665	6594.7474
Canny	77.8953	0.718269498853275	6594.2685
Laplacian	75.7321	0.718206702976486	6594.9691

For a noise-free monochrome image (*I*) of a size (*ι* × *ω*) and its noisy approximation *κ*_(*i*, *j*)_, MSE, PSNR, and SSIM is defined as in Eqs. (4), (5), and (6) respectively.

4 $$ MSE=\frac{1}{\iota \omega}{\sum}_{j=0}^{\iota -1}{\sum}_{j=0}^{\omega -1}{\left[{I}_{\left(i,j\right)}-{\kappa}_{\left(i,j\right)}\right]}^2, $$

5 $$ PSNR=10{\log}_{10}\left(\frac{\gamma^2}{MSE}\right), $$

where *γ* is the maximum information value of the randomness in the given data.

6 $$ SSIM=\left[I\left(\iota, \omega \right)\right]\alpha .\left[\epsilon \left(\iota, \omega \right)\right]\beta .\left[s\left(\iota, \omega \right)\right]\gamma, $$

where, the entries are described as follows: $\alpha =\beta =\gamma =1,\left[I\left(\iota, \omega \right)\right]=\frac{2{\delta}_{\iota }{\delta}_{\omega }+{\epsilon}_1}{{\delta_{\iota}}^2+{\delta_{\omega}}^2+{\epsilon}_1}$, $\epsilon \left(\iota, \omega \right)=\frac{2{\sigma}_{\iota }{\sigma}_{\omega }+{\epsilon}_2}{{\sigma_{\iota}}^2+{\sigma_{\omega}}^2+{\epsilon}_2}$, and $s\left(\iota, \omega \right)=\frac{2{\sigma}_{\iota \omega}+{\epsilon}_3}{\sigma_{\iota \omega}+{\epsilon}_3}$, respectively [53].

*I*(*ι*, *ω*) is a function of luminous comparison to measure the images closeness on the base of mean luminance *δ*_*ι*_ *δ*_*ω*_ of 2-D images *ι and ω*.Maximum value of *I*(*ι*, *ω*) is equal to 1 if and only if *δ*_*ι*_ = *δ*_*ω*_. The second value *ϵ*(*ι*, *ω*) is used to measure the contrast on the base of standard deviation *σ*_*ι*_ and *σ*_*ω*_.The third value *s*(*ι*, *ω*) measures the correlation between two images where *σ*_*ιω*_ is the covariance value. The value of the SSIM lies in the range[0, 1], value *1* shows that two images are determined using the same quality measurement and *0* value indicates no correlation is determined between two mammograms images. According to quality analysis of images after implementing various edge detection techniques: the Sobel and Prewitt operators are considered a good choice. The Prewitt and the Sobel filter are same as filter mask of a 3 × 3 which is used for detection of gradient in the (*x*, *y* ) directions. The only difference exists is the spectral response. Prewitt filter is very suitable for enhancing high frequency and low frequency within the edges of the images edge detection. Sobel operator is a good choice for horizontal borders or edges and Prewitt operator detects better the vertical borders. As pectoral muscle usually appears with vertical border so, Prewitt operation is the best option in the proposed work. It makes use of a 3 × 3 total convolution mask for the detection of gradient (*φ*) in the 2-dimensional case as follows:

7 $$ \varphi =\sqrt{{\varphi_I}^2+{\varphi_{\mathrm{Y}}}^2}, $$

8 $$ \left|\varphi\ \right|=\left|{\varphi}_I\right|+\left|{\varphi}_{\mathrm{Y}}\right|, $$

9 $$ \theta = arrctan\frac{\varphi_I}{\varphi_{\mathrm{Y}}}. $$

Let *I*_*ψ*_ is the image we obtained after label removal, *f*(*φ*) is a function of edge detection implemented on image *I*_*ψ*_ with a threshold.

10 $$ {I}_{\vartheta }={I}_{\psi}\leftarrow f\left(\varphi \right). $$

**Fig. 7:**
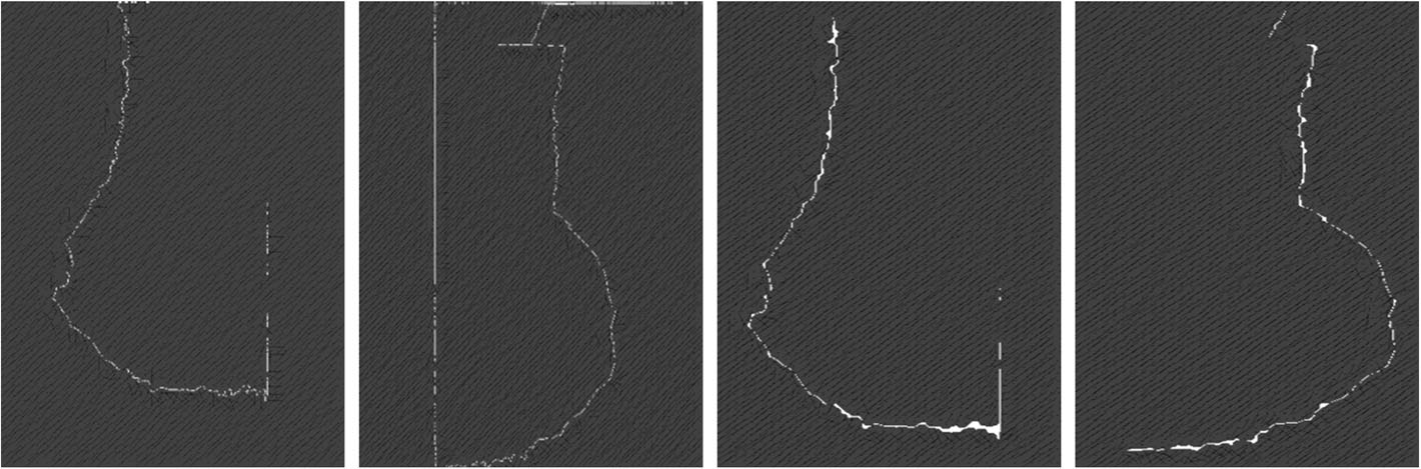
Edges of various mammograms taken by edge detector (Prewitt)

The resultant images (*I*_*ϑ*_) have distorted boundaries as the area where highest intensity variation has been observed, which becomes a part of the background. In this regards, few images are shown in Fig. 7. The output image *I*_*ϑ*_ with broken edges is processed with morphological ‘closing’ operation for obtaining a sealed and accurate boundary. The term ‘closing’ can be defined as a particular background region of a mammogram that is filled with particular color on selective basis. It may be dependent upon an appropriate shaping element of a mammogram for fitting or non-fitting purpose to keep the pectoral muscle structure to be preserved or to be removed. For joining the edges of a broken boundary, morphological closing is used with disk shaped structuring element Ω_*υ*_. Closing is a dual operation of the opening that is produced using the dilation (⨁) of the *I*_*ϑ*_ by Ω_*v*_, followed by the erosion (⊝) as shown in Eq. (11).

11 $$ {I}_{\vartheta}\cdotp {\Omega}_v=\left({I}_{\vartheta}\bigoplus \kern0.5em {\Omega}_v\right)\circleddash {\Omega}_v, $$

where, ${I}_{\vartheta}\bigoplus {\Omega}_v=\bigcup \limits_{b\in B\ }\ {I_{\vartheta}}_b$. Let *f*(*I*_*ϑ*_ · Ω_*v*_) be the closing operation performed on image *I*_*ϑ*_ and the resultant binary image is *I*_*β*_.

12 $$ \kern0.5em {I}_{\beta }={I}_{\vartheta}\leftarrow f\left({I}_{\vartheta}\cdotp {\Omega}_v\right). $$

#### Feature mapping

Convex hull is used in broad-range applications of the computer graphics, CAD, and pattern recognition [37]. In this proposed work, we have used the convex hull to extract the sillhoute of the breast using a topographic map to the binary image. For completing this task, we generate a topographic map (*I*_*σ*_) computing the feature set of four corners for all the foreground pixels in the binary image based on the previous step. A convex hull image (*I*_Δ_) is generated using the map *I*_*σ*_. The *I*_Δ_ has a shape-shifting property. When this image is superimposed on the four corners of the binary image (*I*_*β*_), it shifts the shape according to the map of the binary image and extract the silhouette of the breast body. The resultant image (*I*_δ_) pixels are mapped with original gray scale image for acquiring the segmented breast profile image ( *I*_sτ_) with original intensities of the breast area without pectoral muscles.

13 $$ {I}_{\updelta}\leftarrow {I}_{\Delta}+{I}_{\beta }, $$

14 $$ {I}_{\mathrm{s}\uptau}\leftarrow {I}_{\updelta +}{I}_{\psi }. $$

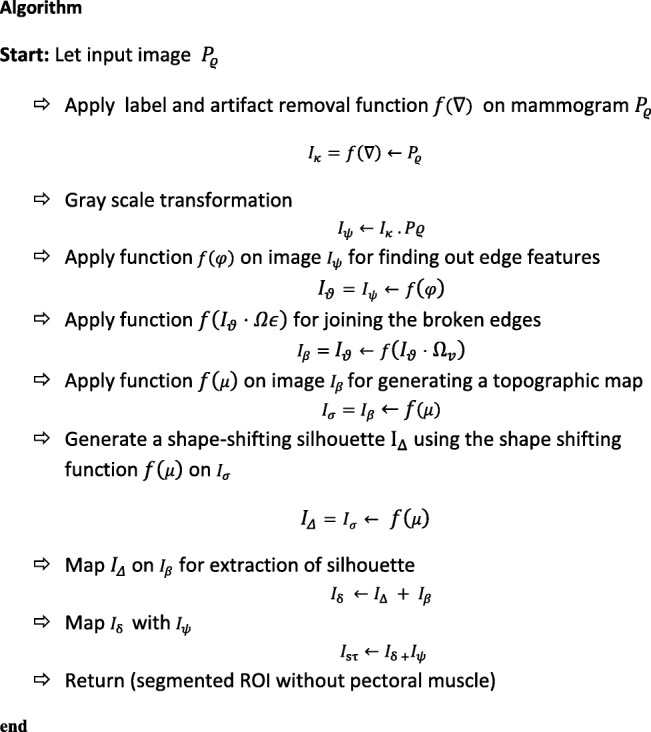

## Experiments and results

**Fig. 8:**
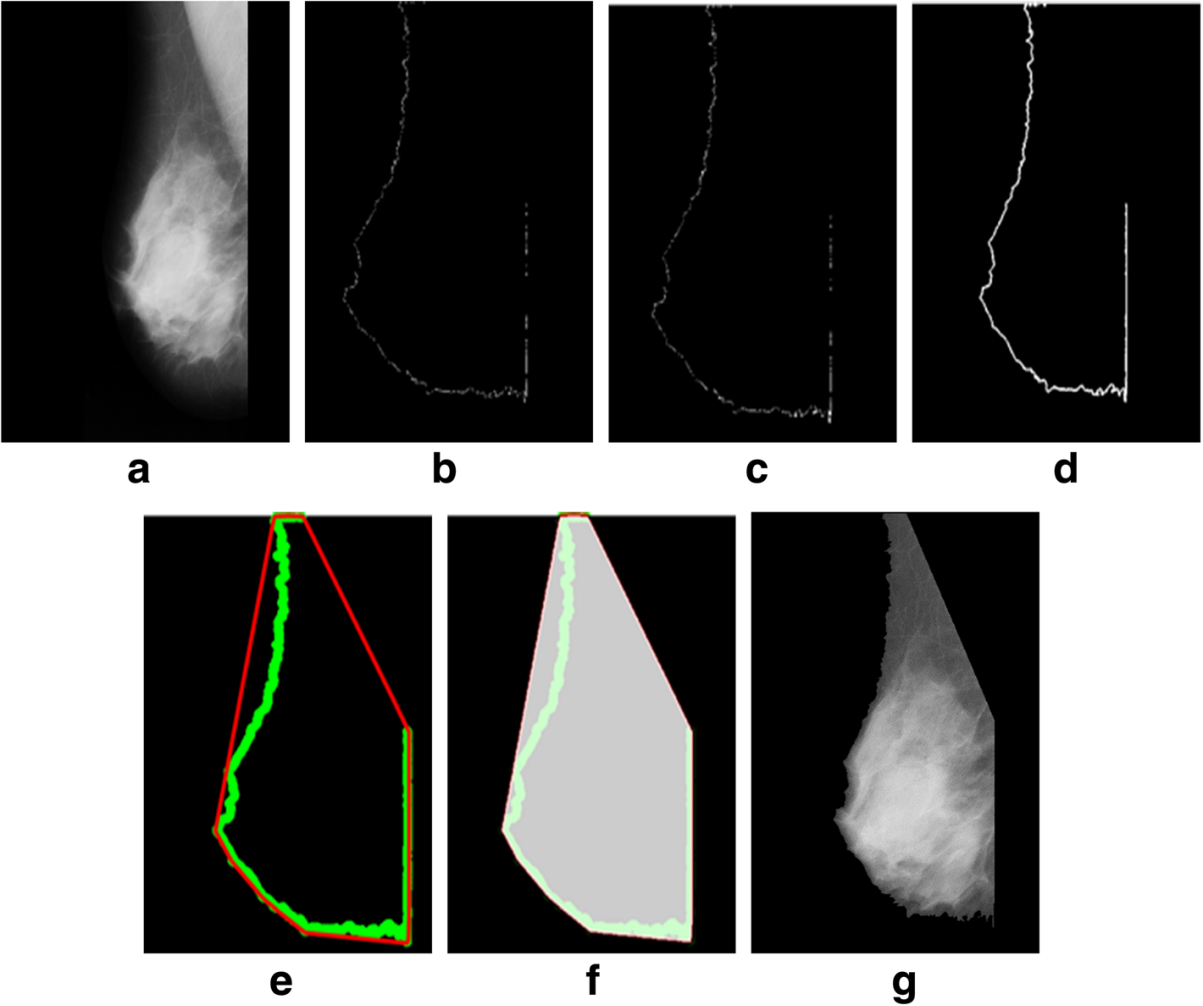
Successful implementation of the proposed algorithm on mdb001 images: **a** original image, **b** edge detection using Prewitt, **c** operation for removing the unnecessary edges, **d** edge smoothness, **e** superimposed the edge pixel for completing the boundary, **f** feature mapping and **g** output image

**Fig. 9:**
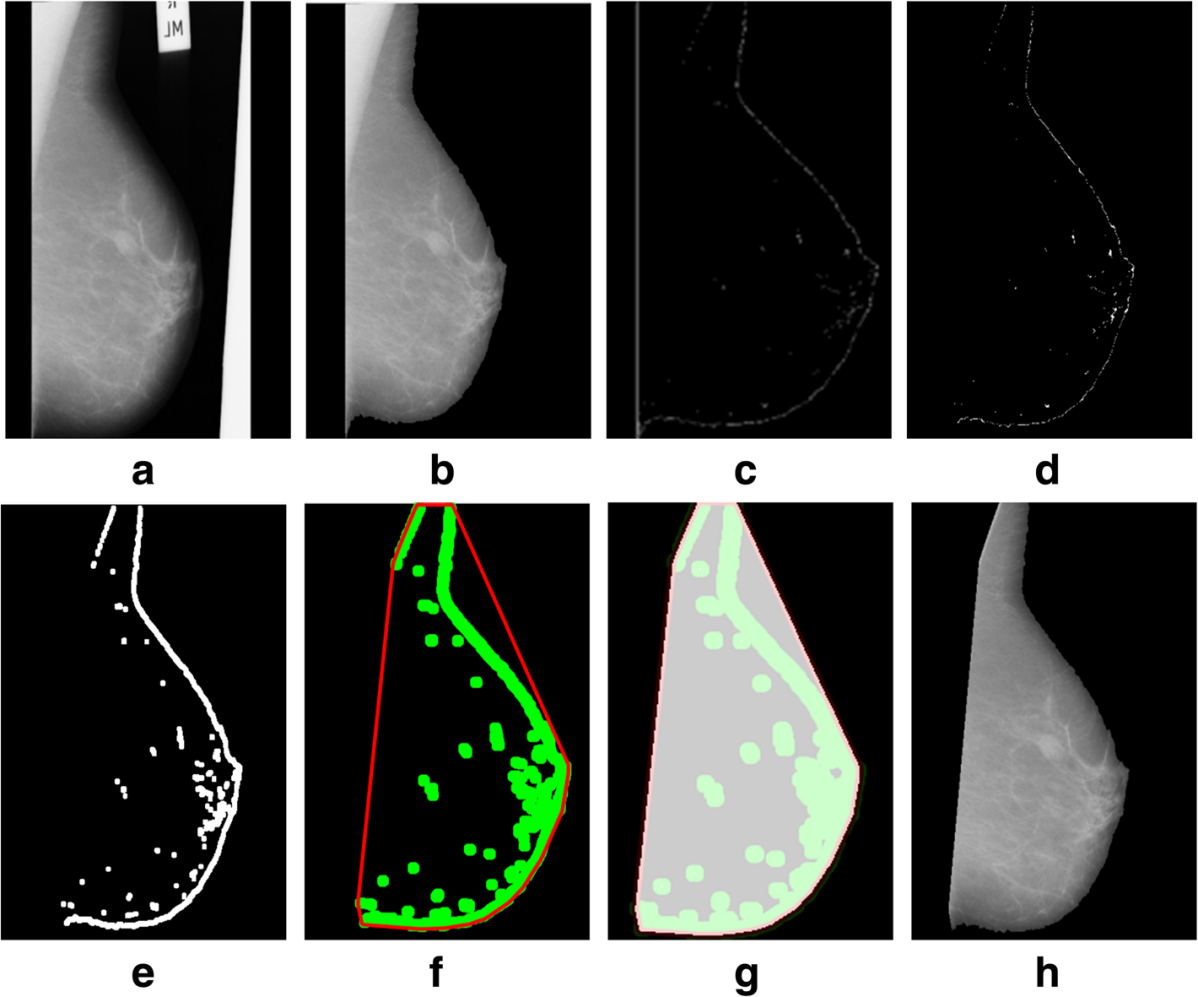
Successful implementation of the proposed method on mdb012 image: **a** given image (original), **b** label removal, **c** edge detection using Prewitt, **d** operation for removing the unnecessary edges, **e** edge smoothness, **f** superimposed the edge pixel for completing the boundary, **g** feature mapping and **h** output image

**Fig. 10:**
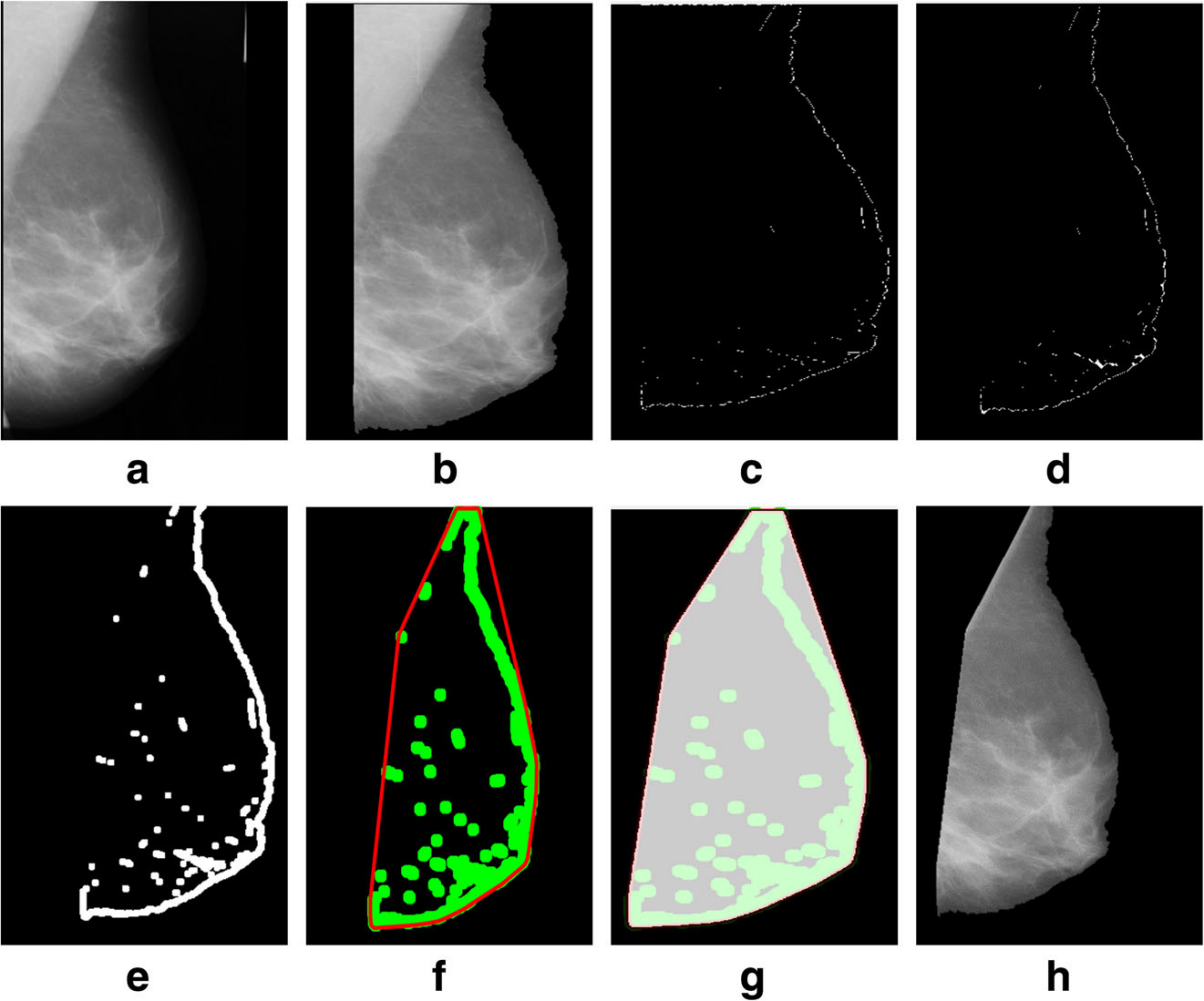
Successful implementation of the proposed algorithm on mdb052 image: **a** original image, **b** label removal, **c** edge detection using Prewitt, **d** operation for removing the unnecessary edges, **e** edge smoothness, **f** superimposed the edge pixel for completing the boundary, **g** feature mapping and **h** output image

**Fig. 11:**
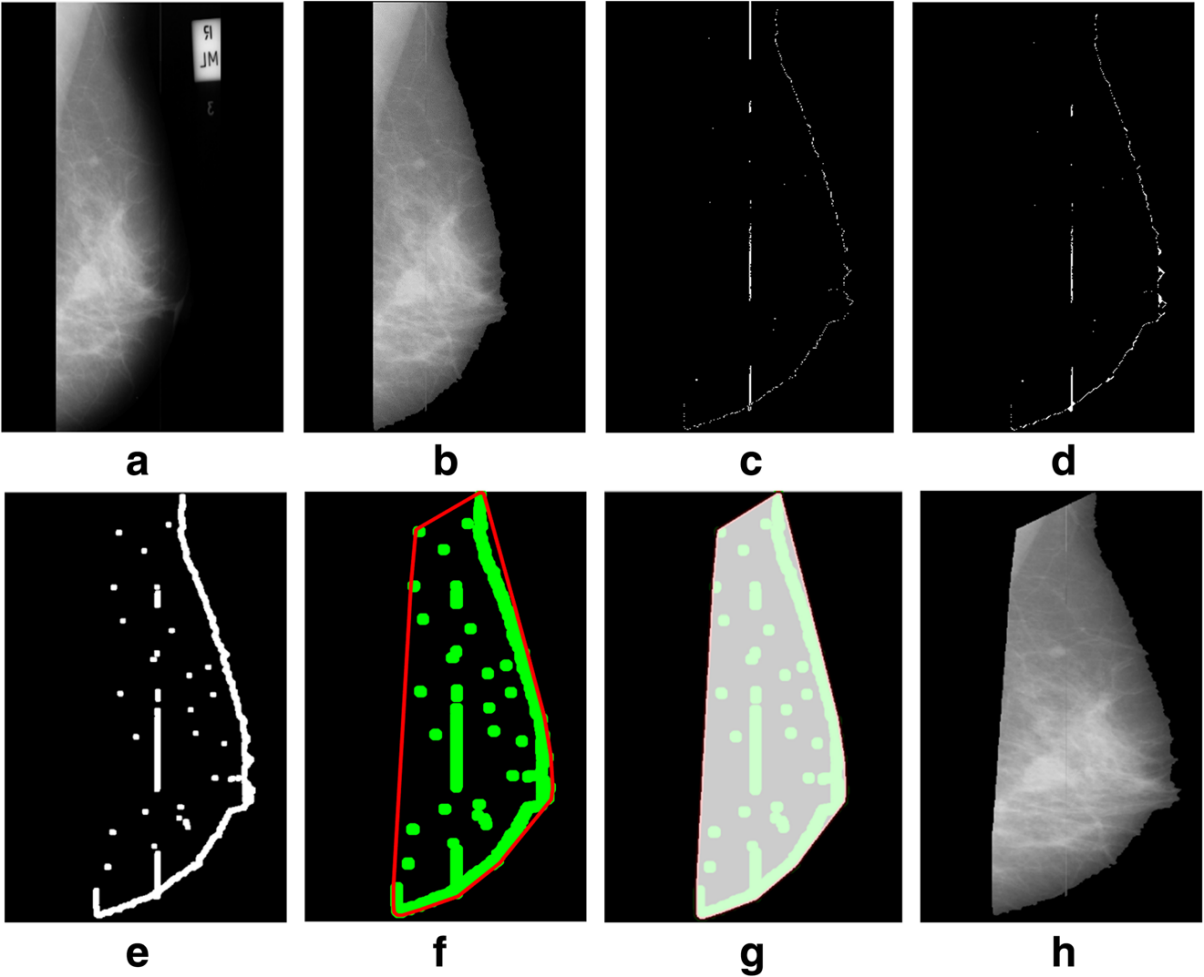
Successful implementation of the proposed algorithm on mdb104 image: **a** original image, **b** label removal, **c** edge detection using Prewitt, **d** operation for removing the unnecessary edges, **e** edge smoothness, **f** superimposed the edge pixel for completing the boundary, **g** feature mapping and **h** output image

**Fig. 12:**
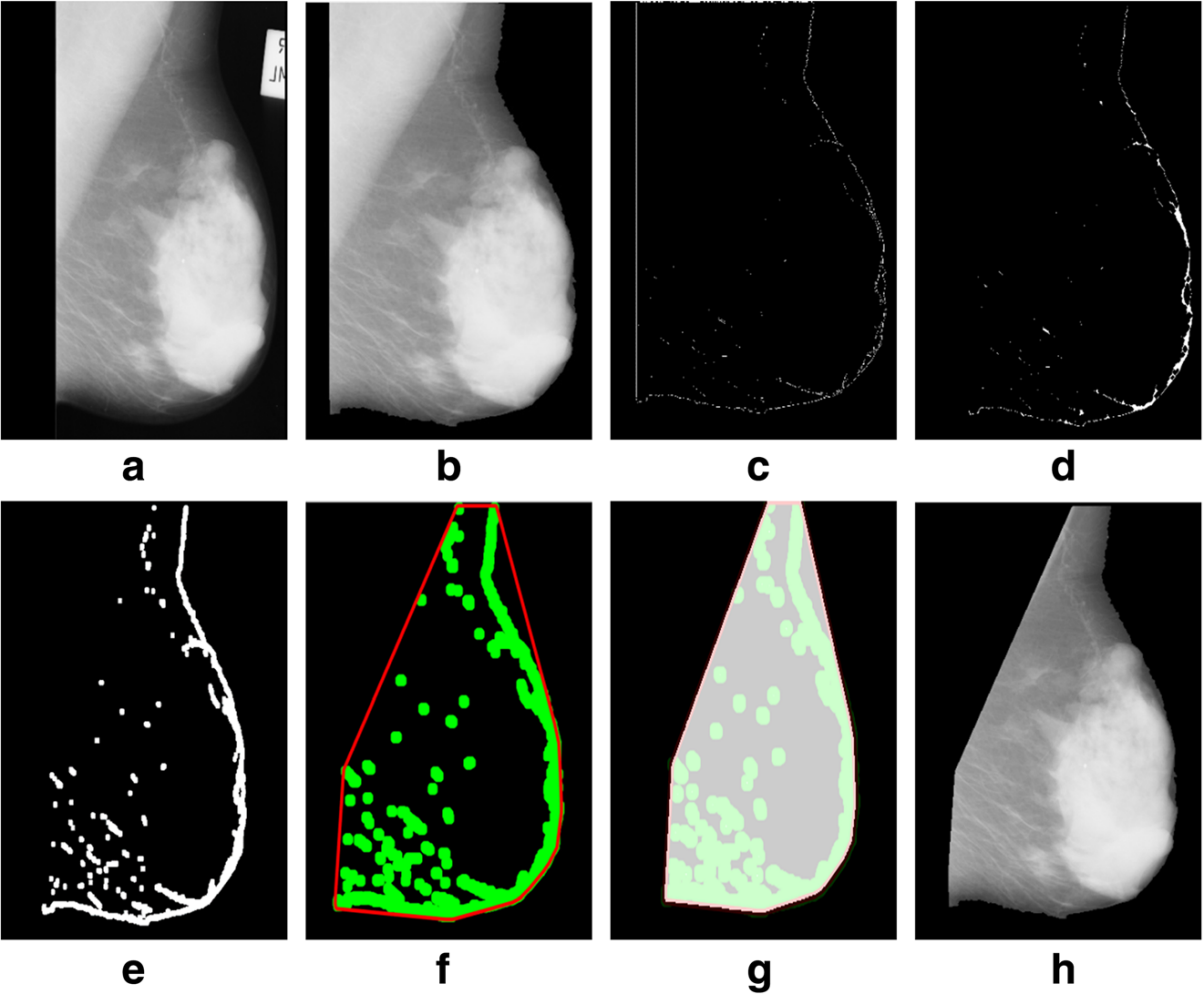
Successful implementation of the proposed algorithm on mdb320 image: **a** original image, **b** label removal, **c** edge detection using Prewitt, **d** operation for removing the unnecessary edges, **e** edge smoothness, **f** superimposed the edge pixel for completing the boundary, **g** feature mapping and **h** output image

**Fig. 13:**
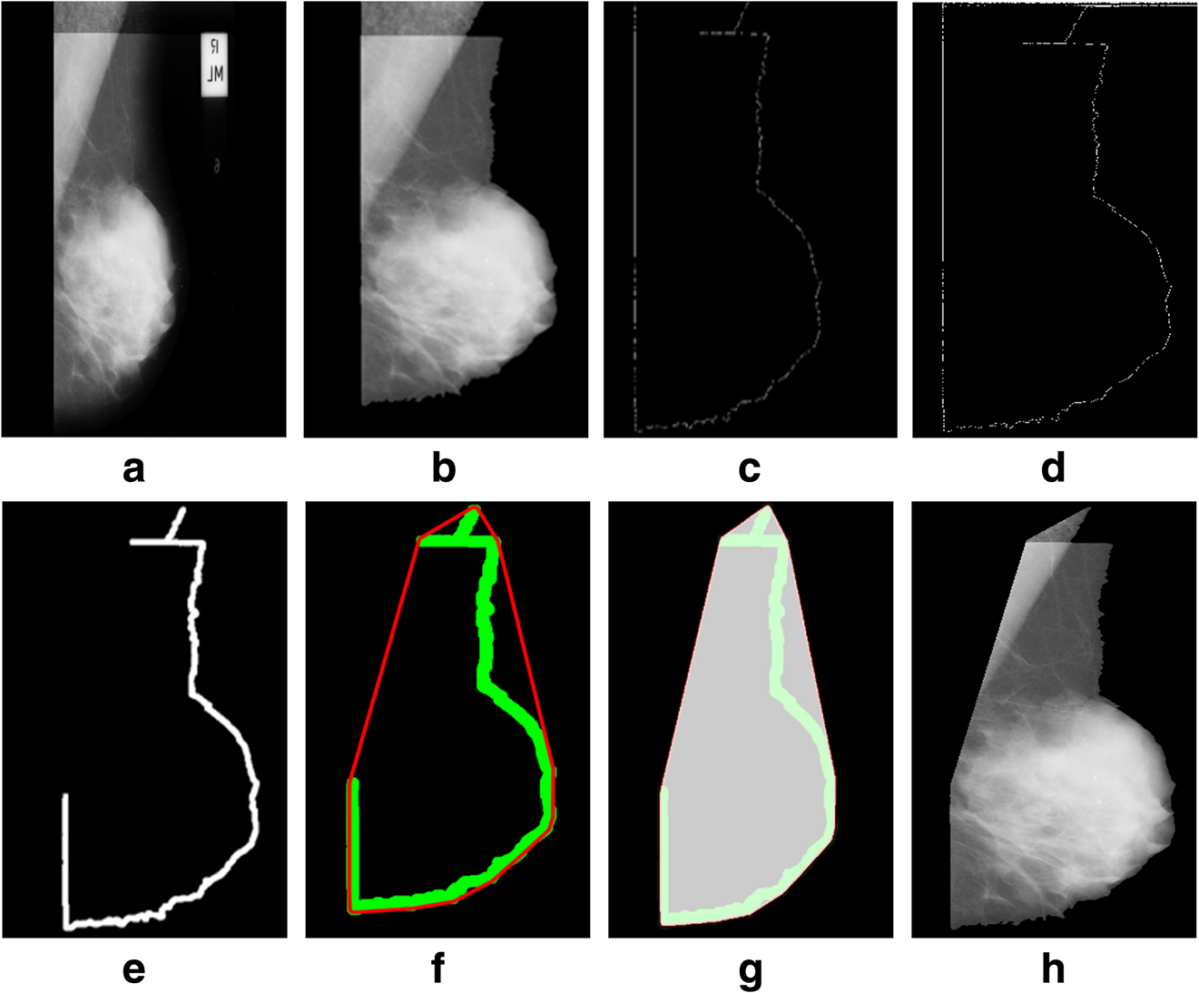
Acceptable implementation of the proposed algorithm on mdb002 image: **a** original image, **b** label removal, **c** edge detection using Prewitt, **d** operation for removing the unnecessary edges, **e** edge smoothness, **f** superimposed the edge pixel for completing the boundary, **g** feature mapping and **h** output image

**Fig. 14:**
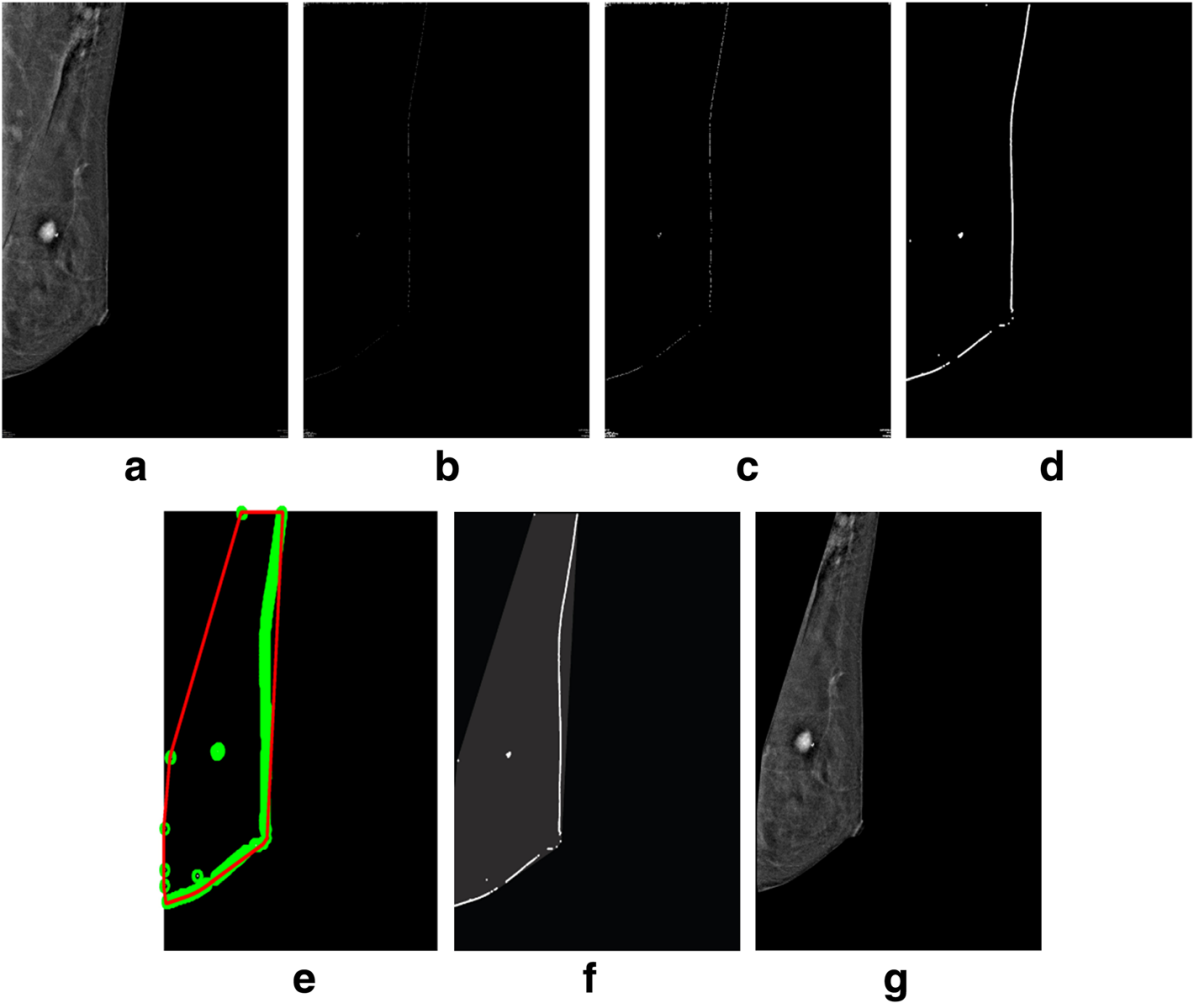
Successful implementation of the proposed algorithm on PT28_CEDMROSE_2013–08-27_LMLO_1.2.840.113681.1377641361.40.1377641361.1 image [38]: **a** original image, **b** edge detection using Prewitt, **c** operation for removing the unnecessary edges, **d** edge smoothness, **e** superimposed the edge pixel for completing the boundary, **f** feature mapping and **g** output image

We tested a mini-MIAS and contrast enhanced digital mammographic images [58–64] to eradicate the pectoral muscle and undesired artifacts. The assessment of the proposed algorithm is done subjectively in two ways; through visual inspection and comparison with a ground truth by an experienced radiologist. According to the first method, the segmentation of a mammogram image can be categorized as follows: successful, acceptable, and unacceptable. Segmentation results are said to be accurate with visible edge information of the entire breast when there are no undesired parts like pectoral muscle is present with breast region as mentioned in Fig. 8. The results are said to be accepted when only some edges of the pectoral muscle remain with breast region. Unaccepted results contain subset of those images that contain half or more than half part of the pectoral muscle in breast mammogram. These results are presented with example in Figs. 8, 9, 10, 11, 12, 13, 14.

### Performance evaluation matrix

A mammogram (*P*_*ϱ*_) is represented using the pixel set *ρ* = {*ρ*_1_, …. *ρ*_*n*_} with |*P*_*ϱ*_| = *row* × *col*; where row is the width and col. is the length of the matrix on which the image is defined. Let the ground truth segmentation provided with data set is represented by ${I}_{g\upomega}^k.$ Moreover, the overlap metrics are defined using the ground truth based segmentation using the partition ${I}_{g\upomega}^k=\left\{{I}_{g\omega}^1,{I}_{g\omega}^2\right\}$ of *P*_*ϱ*_ with assignment function${F}_{\gamma}^{\kappa}\left(\rho \right)$. The segmentation method is performed using the designated algorithm by the partition ${I}_{\mathrm{s}\uptau}=\left\{{I}_{s\tau}^1,{I}_{s\tau}^2\right\}$ of the *P*_*ϱ*_with the assignment function ${F}_{\delta}^i\left(\rho \right)$ that provides the membership of the *ρ* in partition${I}_{s\tau}^{\nu }$. These four basic cardinalities named as *TP*, *TN*, *FP* and *FN* are provided for each pair of a subset $\lambda \in {I}_{g\upvarpi}^k$ and $\eta \in {I}_{s\tau}^{\nu }$. The sum of the weighted value (*ω*_*λη*_) between basic cardinalities is denoted in (15) and Table 2.

15 $$ {\omega}_{\lambda \eta}={\sum}_{h=1}^{P_{\varrho }}{F}_{\gamma}^{\lambda}\left(\rho \right){F}_{\delta}^{\eta}\left(\rho \right),\mathrm{where}={\omega}_{11},\kern0.5em TN={\omega}_{12},\kern0.5em FP={\omega}_{21},\mathrm{and}\  FN={\omega}_{22}. $$

**Table 2:** Confusion matrix

*Confusion Matrix*	${I}_{s\tau}^1$	${I}_{s\tau}^2$
${I}_{g\omega}^1$	*TP*(*ω*_11_)	*TN*(*ω*_12_)
${I}_{g\omega}^2$	*FP*(*ω*_21_)	*FN*(*ω*_22_)

In addition to *TP*(*ω*_11_), *TN*(*ω*_12_), *FP*(*ω*_21_), and *FN*(*ω*_22_), the proposed algorithm is evaluated by measuring the Hausdorff distance. This is used to observe a gap between intensity values *P*_*ϱ*_ based on ground truth data ${I}_{g\upomega}^k$ and intensity values *Pϱ* based on segmented pectoral muscle ${I}_{s\tau}^{\nu }$ is formulated as:

16 $$ HD\left(\ {P}_{\varrho }\ \left({I}_{g\omega}^k\right),{P}_{\varrho}\left({I}_{s\tau}^{\nu}\right)\right)=\mathit{\max}\left(\ \mathit{\min}\ \left( dist\left(\lambda, \eta \right)\right)\right), $$

where, $\lambda \in {I}_{g\omega}^k$ $\kern0.5em and\kern0.5em \eta \in {I}_{s\tau}^{\nu }$, and *dist*(*λ*, *η*) is the Euclidean distance between two points (*λ*, *η*):

17 $$ dist\left(\lambda, \eta \right)=\sqrt{{\left({\lambda}_1-{\eta}_1\right)}^2+\kern0.5em {\left({\lambda}_2-{\eta}_2\right)}^2\ }. $$

Performance of the proposed method is evaluated using all the above discussed performance measures which is presented below in Table 3. Total 322 images are taken from a standard benchmark dataset of the mini-MIAS and 20 images are selected from the contrast enhanced digital mammogram (CEDM) images for evaluating the proposed algorithm.

**Table 3:** Performance measurement of the proposed algorithm

Method	Proposed (MIAS)	Proposed (CEDM)
*FP* mean	0.99	0.98
*FN* mean	5.67	5.66
*FP* < 5 % *and FN* < 5%	57.30	57.25
*Min*(*FP*, *FN*) < 5% 5 % < *Max* (*FP*, *FN*) < 10%	36.50	35.90
*Min* (*FP*, *FN*) < 5% *Max* (*FP*, *FN*) > 10%	3.50	3.48
5 % < *FP* < 10 5 % < *FN* < 10%	0.0	0.0
5 % < *Min* (*FP*, *FN*) < 10% *Max* (*FP*, *FN*) > 10%	1.12	1.15
*FP* > 10 % *and FN* > 10%	0.0	0.0
Hausdorff Distance (HD)	3.52 ± 1.59	3.51 ± 1.58

According to the Hausdorff distance measures, the result obtained using the proposed method shows the smallest mean value 3.51 mm on the CEDM as compared to the MIAS which is 3.52 mm and considered good measurement to remove the pectoral muscle.

## Discussion

Mammograms from the mini MIAS dataset is taken for quantitative evaluation of the proposed method. The rates of *FP*, *FN*, standard deviation, and the mean values of the Hausdorff distance are 0.99, 5.67, 1.59, and 3.52%, respectively. A well-known analysis of the Hough, the Gabor, and the shape based pectoral muscle segmentation methods in comparison with the proposed algorithm are presented in Table 4.

**Table 4:** A brief comparisons of performance of various existing method and the proposed algorithm

Method	[18] Hough	[18] Gabour	[64] Shape based	Proposed (MIAS)	Proposed (CEDM)
*FP* mean	0.58	1.98	1.02	0.99	0.98
*FN* mean	5.77	25.19	5.63	5.67	5.66
*FP* < 5% *FN* < 5%	53.57%	11.90	58.33	57.30	57.25
*Min* (*FP*, *FN*) < 5% 5 % < *Max* (*FP*, *FN*) < 10%	0.0	0.0	35.71	36.50	35.90
*Min* (*FP*, *FN*) < 5% *Max* (*FP*, *FN*) > 10%	0.0	0.0	4.74	3.50	3.48
5 % < *FP* < 10 5 % < *FN* < 10%	26.19%	9.52	0.0	0.0	0.0
5 % < *Min* (*FP*, *FN*) < 10% *Max* (*FP*, *FN*) > 10%	0.0	0.0	1.12	1.12	1.15
*FP* > 10% *FN* > 10%	20.24	78.57	0.0	0.0	0.0
Hausdorff Distance (HD)	3.84 ± 1.73	7.08 ± 5.26	3.53 ± 1.61	3.52 ± 1.59	3.51 ± 1.58

In automated detection of breast tumor detection, the significance of false positive rate is considered more valuable than the false negative rate. However detection results of Gabor filter based and the Hough transform based method in case of both the FP > 10% and the FN > 10% rates are higher than the proposed method. In this method, the Hausdorff distance (HD) is using the designated approach to attain the smallest mean rate as 3.52 mm on the MIAS and 3.51 mm on the CEDM. It is considered to be good measurement to eliminate the unwanted part of the pectoral muscle.

## Conclusion

A novel automatic method is presented for locating the boundary of pectoral muscle. In comparison to the methods shown in the existing literature, the proposed method is not exactly based on straight line detection concept for removing the pectoral muscle. First, differentiation operator is used to detect the edge boundaries and to approximate the gradient value of intensity function. Then, an accurate edge boundary of breast body is determined. Based on the end point of the breast body edges, a convex image is generated. Finally, a convex hull function is developed to produce a topographic map by means of convex image and breast body boundary which is applied on preprocessed mammograms to eradicate the unwanted pectoral muscle. The proposed technique is applied on the benchmark MIAS dataset for a 322 mammograms and a 20 contrasts enhanced digital mammographic images in order to achieve high accuracy in varying size of pectoral muscles.

## Abbreviations

CAD: Computer aided diagnosis
CC: Cranial-caudal
CEDM: Contrast enhanced digital mammogram.
DDSM: Digital database for screening mammography
FN: False negative
FP: False positive
HD: Hausdorff distance
MIAS: Mammographic image analysis society
MLO: Mediolateral-oblique
SE: Structuring element
TP: True positive

## References

[1] Torre LA, Bray F, Siegel RL, Ferlay J, Lortet-Tieulent J, Jemal A (2015). Global cancer statistics, 2012. CA Cancer J Clin.

[2] Tang J, Rangayyan RM, Xu J, El Naqa I, Yang Y (2009). Computer-aided detection and diagnosis of breast cancer with mammography: recent advances. IEEE Trans Inf Technol Biomed.

[3] De Munck L, De Bock G, Otter R, Reiding D, Broeders M, Willemse P, Siesling S (2016). Abstract P6-02-05: digital versus screen-film mammography in population-based breast cancer screening: performance indicators and tumor characteristics of screen-detected and interval cancers. Cancer Res.

[4] Berg WA, Bandos AI, Mendelson EB, Lehrer D, Jong RA, Pisano ED (2016). Ultrasound as the primary screening test for breast cancer: analysis from ACRIN 6666. J Natl Cancer Inst.

[5] Mango VL, Morris EA, Dershaw DD, Abramson A, Fry C, Moskowitz CS, Hughes M, Kaplan J, Jochelson MS (2015). Abbreviated protocol for breast MRI: are multiple sequences needed for cancer detection?. Eur J Radiol.

[6] Bonomi RE, Popov V, Mangner T, Raz A, Shields AF, Gelovani JG (2016). PET imaging of galectin-3 expression with [18F] FPDTG for detection of early breast carcinoma lesions in dense breast tissue. Cancer Res.

[7] Sayed GI, Soliman M, Hassanien AE. Bio-inspired swarm techniques for thermogram breast cancer detection. In: Medical imaging in clinical applications. Springer; 2016. p. 487–506. 10.1007/2F978-3-319-33793-7_21.

[8] He N, Wu Y-P, Kong Y, Lv N, Huang Z-M, Li S, Wang Y, Geng Z-j, Wu P-H, Wei W-D (2016). The utility of breast cone-beam computed tomography, ultrasound, and digital mammography for detecting malignant breast tumors: a prospective study with 212 patients. Eur J Radiol.

[9] Elangeeran M, Ramasamy S, Arumugam K. A novel method for benign and malignant characterization of mammographic microcalcifications employing waveatom features and circular complex valued—extreme learning machine. In: A novel method for benign and malignant characterization of mammographic microcalcifications employing waveatom features and circular complex valued—extreme learning machine. IEEE; 2014. p. 1–6. 10.1109/ISSNIP.2014.6827660.

[10] Liu C-C, Tsai C-Y, Liu J, Yu C-Y, Yu S-S (2012). A pectoral muscle segmentation algorithm for digital mammograms using Otsu thresholding and multiple regression analysis. Comput Math Appl.

[11] Wei C-H, Gwo C-Y, Huang PJ (2016). Identification and segmentation of obscure pectoral muscle in mediolateral oblique mammograms. Br J Radiol.

[12] Mughal B, Sharif M (2017). Automated detection of breast tumor in different imaging modalities: a review. Curr Med Imaging Rev.

[13] Mughal B, Sharif M, Muhammad N (2017). Bi-model processing for early detection of breast tumor in CAD system. Eur Phys J Plus.

[14] Karssemeijer N (1998). Automated classification of parenchymal patterns in mammograms. Phys Med Biol.

[15] Kwok S, Chandrasekhar R, Attikiouzel Y. Automatic pectoral muscle segmentation on mammograms by straight line estimation and cliff detection. IEEE; 2001. p. 67–72. 10.1109/ANZIIS.2001.974051.

[16] de Carvalho IM, Luz L, Alvarenga A, Infantosi A, Pereira W, Azevedo C. An automatic method for delineating the pectoral muscle in mammograms. In: An automatic method for delineating the pectoral muscle in mammograms. Springer; 2007. p. 271–5. 10.1007/978-3-540-74471-9_63.

[17] Mughal B, Muhammad N, Sharif M, Saba T, Rehman A. Extraction of breast border and removal of pectoral muscle in wavelet domain. Biomed Res. 2017;28(11):5041-3.

[18] Ferrari RJ, Rangayyan RM, Desautels JL, Borges R, Frere AF (2004). Automatic identification of the pectoral muscle in mammograms. IEEE Trans Med Imaging.

[19] Mustra M, Bozek J, Grgic M. Breast border extraction and pectoral muscle detection using wavelet decomposition. In: Breast border extraction and pectoral muscle detection using wavelet decomposition. IEEE; 2009. p. 1426–33. 10.1109/EURCON.2009.5167827.

[20] Hoiem D, Efros AA, Hebert M (2007). Recovering surface layout from an image. Int J Comput Vis.

[21] Bajger M, Ma F, Bottema MJ. Minimum spanning trees and active contours for identification of the pectoral muscle in screening mammograms. IEEE; 2005. p. 2005. 10.1109/DICTA.2005.55.

[22] Pobiruchin M, Bochum S, Martens UM, Kieser M, Schramm W (2016). A method for using real world data in breast cancer modeling. J Biomed Inform.

[23] Firmino M, Angelo G, Morais H, Dantas MR, Valentim R (2016). Computer-aided detection (CADe) and diagnosis (CADx) system for lung cancer with likelihood of malignancy. Biomed Eng Online.

[24] Geronimo D, Lopez AM, Sappa AD, Graf T (2010). Survey of pedestrian detection for advanced driver assistance systems. IEEE Trans Pattern Anal Mach Intell.

[25] Abdellatif H, Taha T, Zahran O, Al-Nauimy W, El-Samie FA. K2. Automatic pectoral muscle boundary detection in mammograms using eigenvectors segmentation. IEEE; 2012. p. 633–40. 10.1109/NRSC.2012.6208576.

[26] Domingues I, Cardoso JS, Amaral I, Moreira I, Passarinho P, Santa Comba J, Correia R, Cardoso MJ. Pectoral muscle detection in mammograms based on the shortest path with endpoints learnt by SVMs. IEEE; 2010. p. 3158–61. 10.1109/IEMBS.2010.5627168. 10.1109/IEMBS.2010.5627168 21096595

[27] Wang L, Zhu M-l, Deng L-p, Yuan X (2010). Automatic pectoral muscle boundary detection in mammograms based on Markov chain and active contour model. J Zhejiang Univ Sci C.

[28] Rouhi R, Jafari M, Kasaei S, Keshavarzian P (2015). Benign and malignant breast tumors classification based on region growing and CNN segmentation. Expert Syst Appl.

[29] Naseer A, Daniele L, Muhammad N, Cristiano C, Guido F, Livio SB, Mebratu AG, Aslam M, Giovanni BL, Giuseppe F (2017). Sphingosine 1-phosphate receptor modulator fingolimod (Fty720) attenuates myocardial fibrosis in post-heterotopic heart transplantation. Front Pharmacol.

[30] Sultana A, Ciuc M, Strungaru R. Detection of pectoral muscle in mammograms using a mean-shift segmentation approach. In: Detection of pectoral muscle in mammograms using a mean-shift segmentation approach: IEEE; 2010. p. 165–8. 10.1109/ICCOMM.2010.5509003.

[31] Muhammad N, Sharif M, Jaweria A, Riffat M, Nargis B, Naseer A (2018). Neurochemical alterations in sudden unexplained perinatal deaths-a-review. Front Pediatr.

[32] Muhammad N, Bibi N, Mahmood Z, Kim DG (2015). Blind data hiding technique using the Fresnelet transform. Springerplus.

[33] Muhammad N, Bibi N, Mahmood Z, Akram T, Naqvi SR (2017). Reversible integer wavelet transform for blind image hiding method. PLoS One.

[34] Saltanat N, Hossain MA, Alam MS. An efficient pixel value based mapping scheme to delineate pectoral muscle from mammograms. In: An efficient pixel value based mapping scheme to delineate pectoral muscle from mammograms: IEEE; 2010. p. 1510–7. 10.1109/BICTA.2010.5645272.

[35] Aslam A, Bashir Y, Rafiq M, Haider F, Muhammad N, Bibi N. Three New/Old Vertex-Degree-Based Topological Indices of Some Dendrimers Structure. Electronic J Biol. 2017;13(1):94-9.

[36] Yasir B, Adnan A, Muhammad K, Muhammad IQ, Adnan J, Muhammad R, Nargis B, Nazeer M (2017). On forgotten topological indices of some dendrimers structure. Molecules.

[37] Hong B-W, Sohn B-S (2010). Segmentation of regions of interest in mammograms in a topographic approach. IEEE Trans Inf Technol Biomed.

[38] Shabieh F, Nazeer M, Tariq S, Sohail A (2017). A novel image encryption based on algebraic S-box and arnold transform. 3D Res.

[39] Muhammad N, Nargis B (2015). Digital image watermarking using partial pivoting lower and upper triangular decomposition into the wavelet domain. IET Image Process.

[40] Muhammad N, Nargis B, Adnan J, Zahid M. Image denoising with norm weighted fusion estimators. Pattern Anal Applic. 2017:1–10. 10.1007/s10044-017-0617-8.

[41] Muhammad N, Nargis B, Iqbal Q, Adnan J, Zahid M. Digital watermarking using hall property image decomposition method. Pattern Anal Applic. 2017:1–16. 10.1007/s10044-017-0613-z.

[42] Mirzaalian H, Ahmadzadeh M, Sadri S. Pectoral muscle segmentation on digital mammograms by nonlinear diffusion filtering. In: Pectoral muscle segmentation on digital mammograms by nonlinear diffusion filtering: IEEE; 2007. p. 581–4. 10.1109/PACRIM.2007.4313303.

[43] Goodsitt MM, Chan HP, Liu B, Guru SV, Morton A, Keshavmurthy S, Petrick N (1998). Classification of compressed breast shapes for the design of equalization filters in x-ray mammography. Med Phys.

[44] Nargis B, Anthony K, Muhammad N, Barry C (2016). Equation-method for correcting clipping errors in Ofdm signals. SpringerPlus.

[45] Nargis B, Muhammad N, Barry C. Inverted wrap-around limiting with Bussgang noise cancellation receiver for Ofdm signals. Circuits Syst Signal Process. 2017:1–14.

[46] O'Connor JPB, Tofts PS, Miles KA, Parkes LM, Thompson G, Jackson A. Dynamic contrast-enhanced imaging techniques: CT and MRI. Brit J Radiol. 2011;84 (special_issue_2):S112-S20. PubMed PMID: 22433822. 10.1259/bjr/55166688 PMC3473907 22433822

[47] Muhammad N, Bibi N, Wahab A, Mahmood Z, Akram T, Naqvi SR, Oh HS, Kim DG. Image de-noising with subband replacement and fusion process using bayes estimators. Comput Electr Eng. 2017. 10.1016/j.compeleceng.2017.05.023.

[48] Khan MA, Akram T, Sharif M, Javed MY, Muhammad N, Yasmin M. An implementation of optimized framework for action classification using multilayers neural network on selected fused features. Pattern Anal Applic. 2018. 10.1007/s10044-018-0688-1.

[49] Kim NM, Dai-Gyoung (2013). Resolution enhancement for digital off-Axis hologram Reconstruction. Iaeng transactions on engineering technologies.

[50] Mahmood Z, Ali T, Muhammad N, Bibi N, Shahzad I, Azmat S (2017). EAR: Enhanced Augmented Reality system for sports entertainment applications.

[51] Mahmood Z, Muhammad N, Bibi N, Ali T (2017). A review on state-of-the-art face recognition approaches. Fractals.

[52] Wang Z, Bovik AC, Sheikh HR, Simoncelli EP (2004). Image quality assessment: from error visibility to structural similarity. IEEE Trans Image Process.

[53] Shabieh F, Shah T, Muhammad N, Bibi N, Jahangir A, Arshad S (2017). An image encryption technique based on chaotic S-box and Arnold transform. Int J Adv Comput Sci Appl.

[54] Bhateja V, Misra M, Urooj S (2016). Non-linear polynomial filters for edge enhancement of mammogram lesions. Comput Methods Prog Biomed.

[55] Naqvi SR, Akram T, Iqbal S, Haider SA, Kamran M, Muhammad N (2018). A dynamically reconfigurable logic cell: from artificial neural networks to quantum-dot cellular automata. Appl Nanosci.

[56] Irshad M, Muhammad N, Sharif M, Yasmeen M (2018). Automatic segmentation of the left ventricle in a cardiac MR short axis image using blind morphological operation. Eur Phys J Plus.

[57] Mughal B, Sharif M, Muhammad N, Saba T. A novel classification scheme to decline the mortality rate among women due to breast tumor. Microsc Res Tech. 2018;81(2):171-80. 10.1002/jemt.22961 29143395

[58] Jochelson M (2014). Contrast-enhanced digital mammography. Radiol Clin N Am.

[59] Baljeet S, Samreen A, Adnan J, Muhammad N (2017). Plane harmonic waves in rotating medium under the effect of micro-temperature and dual-phase-lag thermoelasticity. UPB Sci Bull Ser D.

[60] Usman M, Saba K, Han D-P, Muhammad N. Efficiency improvement of green light-emitting diodes by employing all-quaternary active region and electron-blocking layer. Superlattice Microstruct. 2018;1(113):585-91.

[61] Muhammad U, Kiran S, Dong-Pyo H, Muhammad N, Shabieh F, Rafiqu M, Tanzila S (2018). AIP Adv.

[62] Abo-Dahab SM, Jahangir A, Muhammad N, Farwa S, Bashir Y, Usman M. Propagation phenomena in a visco-thermo-micropolar elastic medium under the effect of micro-temperature. Results Phys. 2018;8:793-8.

[63] Atwa SY, Nazeer M, Adnan J, Rehman N (2017). Influence of energy dissipation on plane harmonic waves through a piezo-thermo-elastic medium. Eur Phys J Plus.

[64] Chen C, Liu G, Wang J, Sudlow G (2015). Shape-based automatic detection of pectoral muscle boundary in mammograms. J Med Biol Eng.

